# The triple whammy anxiety depression and osteoarthritis in long-term conditions

**DOI:** 10.1186/s12875-015-0346-2

**Published:** 2015-10-14

**Authors:** Valerie Tan, Clare Jinks, Carolyn Chew-Graham, Emma L. Healey, Christian Mallen

**Affiliations:** Arthritis Research UK Primary Care Centre, Research Institute for Primary Care Centre, Keele University, Staffordshire, ST5 5BG UK

**Keywords:** Anxiety, Depression, Osteoarthritis, Long-term conditions, Case-finding, Integrated care

## Abstract

Improving the management of people with long-term conditions is a key priority of the UK National Health Service. Whilst the coexistence of two or more long-term conditions in one person is increasingly the norm in primary care, guidelines and delivery of care remain focused on single disease management.

Anxiety, depression and osteoarthritis are frequently comorbid with other long-term conditions and with each other, with up to 70 % of people with anxiety and depression also suffering from chronic pain. The relationships between anxiety, depression and pain are reciprocal, with each predicting and worsening the outcome of the others. Where these conditions occur in the context of other long-term conditions, further reduction in health-related quality of life and poorer clinical outcomes for all comorbid conditions is observed. It therefore follows that optimising the management of one comorbid condition should confer benefit to the other/s. Yet despite this, anxiety, depression and chronic pain are seldom prioritised by either patient or clinician, therefore remaining under-recognised and under-treated.

Case-finding aims to identify and offer timely treatment to individuals with a given disease in a population at risk, therefore offering one possible solution. Yet case-finding is not without its problems, with well-recognised barriers including lack of time, cultural difficulties and inadequate resources and practitioner skills. So whilst the merits of why to actively seek these conditions is clear, how this may be best achieved is not. We explore the potential role of case-finding for anxiety, depression and osteoarthritis-related joint pain in individuals with comorbid long-term conditions, assessing whether adopting an integrated approach to care may allow opportunistic case-finding therefore promoting identification and timely management of these deleterious conditions.

## Background

Improving the management of people with long-term conditions (LTCs) has been a key priority of the UK National Health Service (NHS) for over 20 years [1]. Indeed, a global need for change in the way we care for patients with LTCs led to the development of the Chronic Care Model in the late 1990’s [2]. Yet guidelines and delivery of care typically remain focused on single disease management, whilst the coexistence of two or more LTCs in one person is increasingly the norm in primary care [3].

Anxiety and depression are common in people with LTCs, with rates two-to-three times greater in patients with chronic physical illness compared with their age- and gender-matched peers [4]. Likewise osteoarthritis (OA), the most prevalent cause of chronic pain in older adults, is often comorbid with other LTCs [5]. Anxiety, depression and OA-related joint pain frequently coexist, with up to 70 % of people with anxiety and depression also suffering from chronic pain [6]. Thus the ‘triple whammy’ of LTC with anxiety/depression and chronic pain is a common occurrence. Yet whilst management of LTCs has gained much impetus over the last two decades; anxiety, depression and chronic pain remain under-prioritised, under-detected and under-treated. Could opportunistic case-finding for patients with such comorbidity offer a viable solution?

The relationships between anxiety, depression and pain are reciprocal, with each predicting and worsening the outcome of the others [6]. Where these conditions occur in the context of other LTCs, further reduction in health-related quality of life and poorer clinical outcomes for all comorbid conditions is observed. Patients with comorbid depression and diabetes or coronary heart disease (CHD), two of the most prevalent LTCs in primary care, frequently have poorer self-care, increased symptom burden, greater level of functional impairment and increased risk of both disease complications and mortality [7]. It therefore follows that optimising the management of one comorbid condition should confer benefit to the other/s, with interventions targeted at specific combinations of common conditions potentially conferring the most benefit [8]. Indeed, identifying and managing comorbid depression by adopting an integrated approach to the care of patients with coexistent diabetes or CHD results in significant improvement in both medical and psychological outcomes [9]. Despite this evidence, in the context of comorbidity, anxiety, depression and chronic pain are seldom prioritised by either patient or clinician, both engaging in attributional styles that normalise symptoms as an understandable reaction to chronic disease and to be expected [10]. As such anxiety, depression and chronic pain are often under-recognised and under-treated.

Whilst the merits of *why* clinicians should identify and validate a patient’s symptoms may be apparent, the *how* is not so clear. One method of improving detection rates is through active case-finding; the strategic screening of a given group of individuals with the specific objective of detecting disease and subsequently offering treatment [11]. The need to proactively seek symptoms of depression in people with LTCs has long been recognised in both the academic literature and clinical guidelines with the National Institute for Health and Care Excellence (NICE) publishing specific guidance outlining the evidence-based treatment of depression in adults with a chronic physical health problem in 2009 [12]. Indeed until 2013, case-finding for depression was incentivised through inclusion in the Quality and Outcomes Framework (QOF) component of the general practice (GP) contract, the incentive programme detailing GP practice achievement results. However despite widespread recognition of unmet patient need, incentivised case-finding failed to result in measurable patient benefit; encouraging a prescriptive, out-of-context assessment contrary to individualised patient-centred care [13]. Incentivised case-finding for depression in primary care was consequently withdrawn. Yet whilst incentivised case-finding for depression proved to be of little benefit, withdrawal from QOF served to foster the well documented and often dysfunctional relationship in which the practitioner is reluctant and patient reticent to discuss depression [10]; hence the unmet need of the patient persists.

Anxiety symptoms are more common in the community than those of depression [14] yet case-finding for anxiety has gained far less attention. Similarly whilst there is widespread recognition of the prevalence and impact of comorbid OA-related joint pain, clinical guidance and management protocols are notable for their lack of consideration of case-finding.

Many of the barriers to case-finding for anxiety and OA-related joint pain in primary care are common to those for other conditions including lack of time, cultural barriers and inadequate resources and practitioner skills [10]. However, clinicians may additionally question the appropriateness of case-finding for anxiety due to the lack of evidence of effectiveness and acceptability of case-finding tools, often complaining that such schedules are logistically problematic to use in a patient-centred consultation [14].

With regards OA-related joint pain; the lack of a clear, singular definition for OA has resulted in differences in the threshold for diagnosis, for some precluding active case-finding in primary care. However whilst it is true that OA can be defined objectively based on the presence of structural joint pathology or more subjectively based on patient-reported symptoms; both the World Health Organisation (WHO) and NICE actively endorse clinical diagnosis based on patient reported symptoms alone without recourse to investigation [15, 16], potentially allaying such concerns.

The purpose of case-finding is to identify and offer timely treatment to individuals with a given disease in a population at risk [11]. As such, an essential component of case-finding is the provision of an effective treatment or intervention for those identified. Whilst NICE guidance outlines the evidence-based management of both anxiety and OA, the lack of appropriate, readily available support and treatment including adequate provision of local services may also result in reluctance to engage in case-finding. Indeed with regards to chronic pain, authors of the National Pain Audit 2012 recognised the lack of agreed standards of care and clear variation in provision of service [17]. Furthermore, primary care clinicians may have limited capacity to support patients with newly recognised anxiety, depression or joint pain. Patients and their caregivers may also have limited capacity to manage an increase in treatment burden imposed as a result of a new label acquire through case-finding, with consequent demands of treatment potentially, and somewhat paradoxically, having a negative impact on their behavioural, cognitive, physical and psychosocial well-being [18].

## Conclusions

Whatever the barriers, untreated anxiety, depression and OA-related joint pain will adversely affect the clinical outcomes and prognosis of patients with LTCs. Change is therefore required on two fronts. Firstly, we must change the perception of these conditions as inevitable and ‘normal’ thus empowering both patients and clinicians to implement change; facilitating effective, patient-centred dialogue within the primary care consultation.

Secondly, these changes must be supported by the reconfiguration of existing service delivery models and targeted commissioning of services such as described in ‘the house of care’ [19]. Adopting an integrated approach to care may allow effective, opportunistic case-finding and ensure provision of appropriate management of both physical and mental health problems thereby facilitating identification and timely management of these pervasive and deleterious conditions.

## Abbreviations

CHD: Coronary heart disease
GP: General practice
LTC: Long-term condition
NHS: National Health Service
NICE: National Institute for Health and Care Excellence
OA: Osteoarthritis
QOF: Quality and Outcomes Framework
WHO: World Health Organisation
